# Effect of psychosocial motivations and technology on physical activity behaviours among community older men and women

**DOI:** 10.1186/s12877-022-03654-8

**Published:** 2022-12-03

**Authors:** Yong Lin Lee, Gina S. Lee, Louis LY Teo, Ru-San Tan, Liang Zhong, Fei Gao, Angela S. Koh

**Affiliations:** 1 Lee Kong Chian School of Medicine, Nanyang Technological University, Singapore, Singapore; 2 National Heart Centre Singapore, 5 Hospital Drive, 169609 Singapore, Singapore; 3 Duke-NUS Medical School, Singapore, Singapore

**Keywords:** Exercise, Psychosocial, Cardiovascular, Digital health

## Abstract

**Background:**

Implementation of physical activity strategies in older populations may be influenced by underlying psychosocial and gender-based factors to physical activity. We explored associations between these factors and physical activity behaviors and technology among older men and women.

**Methods:**

Community older adults underwent echocardiography and interviewer administered questionnaires that collected physical activity habits, self-motivation, self-empowerment and smartphone usage patterns associated with physical activity. Aerobic capacity was denoted by VO_2_max (High VO_2_ was defined as VO_2_ > 35 (ml/kg/min) for men or VO_2_ > 27 (ml/kg/min) for women).

**Results:**

Among 180 participants (mean age 77 (71–80) years; 43% females), 101 (56.1%) had a low VO_2_max. Barriers to activity were lack of time (27.8%), tiredness (26.7%), affordability (12.8%) and pain while exercising (12.2%). Compared to participants with high VO_2_max, those with low VO_2_max were less likely to report feeling good post-exercise (70.3% vs 86.1%, adjusted *p* = 0.041) and express barriers to exercise (72.3% vs 88.6%, adjusted *p* = 0.017). Compared to men, women were more likely to express motivation for exercise if they were guided by an instructor (20.5% vs 1.96%, adjusted *p* = 0.027), less likely to prefer control over exercise type and difficulty (57.7% vs 82.4%, adjusted *p* = 0.001), express interest in smartphone apps (7.84% vs 24.4%, adjusted *p* *=* 0.01) and participate in apps-guided exercise (10.3% vs 29.4%, adjusted *p* = 0.001). Major factors that motivated the use of smartphone applications to manage individual health were financial incentives (23.9%) and guidance on exercise routines (21.1%) while the reveal of personal information was a major deterrent (28.3%).

**Conclusions:**

We observed differences in physical activity motivation, empowerment and technology use based on gender and functional status. Tailoring physical activity strategies, including digital health strategies, that target psychosocial and gender-based factors may improve activity participation in older adults.

## Introduction

Cardiorespiratory fitness measures an individual’s maximum aerobic capacity [1] and has been shown to be a strong predictor of heart disease and all-cause mortality [2]. Although there are many determinants of cardiorespiratory fitness, exercise/physical activity has demonstrated strong associations with cardiorespiratory fitness [3], particularly with aerobic capacity.

One of the key components of healthy ageing is being able to function independently [4] and to be free of disease-specific symptoms [5]. Ageing itself is associated with numerous changes such as a decline in aerobic capacity [6] and such changes predispose the older population to the development of cardiovascular disease [7]. On the molecular level, age-related modifications in mitochondrial function have been proven to have detrimental effects on numerous systems including the cardiorespiratory and musculoskeletal systems [8]. Hence numerous guidelines advocating the benefits of physical activity have been released in recent years to promote healthy ageing [9, 10]. In addition to its effects on cardiovascular health, regular physical activity also minimizes age-related losses in skeletal muscle mass and strength [11], hence improving quality of life [12]. Other studies have also shown the positive impact of nutritional modifications and various types of exercise on mitochondrial function and antioxidant capacity, demonstrating the potential of non-pharmacological interventions as a counter to mitochondrial ageing [8, 13]. Despite the importance of physical activity as a contributor to healthy ageing, levels of physical inactivity remain high [14, 15], suggesting a need to increase participation in physical activity through other means.

Previous studies have suggested that physical health, psychological motivations and personal achievements are key motivators for regular participation in exercise [16–18]. Other studies have cited extrinsic factors such as accessibility to facilities and weather as well as intrinsic barriers such as health and disinterest as reasons for poor participation in regular exercise [15, 17, 19]. However, the long-term impact of these motivations and barriers to regular exercise on cardiovascular health in older adults is still poorly understood. An understanding of these psychosocial factors would be key to implementing effective strategies to improve cardiorespiratory fitness at the population level, hence reducing the burden of cardiovascular diseases on healthcare systems.

This study aims to investigate the association between psychosocial factors such as motivation, self-empowerment, including patterns of smartphone usage, with aerobic capacity, in community older adults. We also explored whether gender differences exist between these psychosocial factors.

## Methods

### Patients and study design

The subjects were recruited from the prospective, population-based cohort, The Cardiac Ageing Study (CAS) [20–22], that examines characteristics and determinants of cardiovascular function in older adults over time. This single centre study sample consisted of men and women who participated in the baseline CAS 2014 examination and had returned for longitudinal follow-up of their cardiovascular health status examinations in 2019–2020. They did not have history of physician-diagnosed cardiovascular disease (such as coronary heart disease, stroke) or cancer. We did not apply age-specific exclusions and analysed all subjects who completed the evaluations within the studied time frame. This analysis was a cross-sectional analysis of their cardiovascular function and psychosocial factors. Informed consent was obtained from participants upon enrolment. The institutional review board had approved the study protocol (Institutional Review Board: CIRB 2019/2252). The study was performed in a single study site at the National Heart Centre Singapore.

Within one study visit, clinical data were obtained simultaneously as assessment of echocardiography and questionnaires, by trained imaging operators and study coordinators respectively. Clinical data such as medical history and coronary risk factors were obtained through a standardized questionnaire. Dyslipidaemia was defined by the current use of lipid-lowering agents or physician-diagnosed dyslipidaemia. Hypertension was defined by the current use of antihypertensive drugs or physician-diagnosed hypertension. Diabetes mellitus was defined by the current use of antidiabetic agents or physician-diagnosed diabetes mellitus. Smoking history was defined as ever smokers (former or current smoking) or never smokers. Alcohol history was defined as ever drinks (current or past drinkers) or never drinkers, where drinkers were defined as those who consumed alcoholic beverages once a month or more frequently. Body mass index was calculated as weight in kilograms divided by the square of height in meters. Sinus rhythm status was ascertained by a resting electrocardiogram. Waist circumference was obtained 2.5 cm above the umbilicus [23].

A validated non-exercise prediction model was used to estimate peak oxygen uptake, VO_2_ milliliter/kg/minute (ml/kg/min) [24, 25]. The physical activity questionnaire included frequency of physical activity (inactive, once a week, two to three times a week, almost everyday), length of time for each activity (less than 15 minutes, 16–30 minutes, > 30–60 minutes, >one hour) and intensity of each activity (‘I take it easy without breathing hard or sweating’, ‘little hard breathing or sweating’, ‘near exhaustion’). Physical activity is defined as any bodily movement that requires energy expenditure and varied between aerobic, anaerobic or combined activities for each participant. The VO_2_ calculator is available online (https://www.worldfitnesslevel.org). This model was shown in a previous study to be closely linked to specific measures of cardiovascular structure and function [22]. The authors reviewed the literature related to physical fitness and exercise as well as discussed the questions with members of the research team. A structured pre-specified set of questions was developed after literature review with modification. Participants were asked a series of questions that relate to motivations behind their physical activity, barriers toward physical activity and smartphone usage (questions are indicated in Tables 2 and 3). The questions were administered to participants via face-to-face interviews.

### Instruments

Echocardiography was performed using ALOKA α10 (Hitachi Medical, Wallington, CT, USA) with a 3.5-MHz probe. In each subject, standard echocardiography, which included 2-D, M-mode, pulse Doppler, and tissue Doppler imaging, was performed in the standard parasternal and apical (apical 4-chamber, apical 2-chamber, and apical long) views, and three cardiac cycles were recorded. Left ventricular ejection fraction, left atrial (LA) volume, and LA volume index were measured. The trans-mitral flow E and A wave with the sample volume position at the tip of the mitral valve leaflets from the apical 4-chamber view were recorded by Doppler echocardiography. Pulsed wave tissue Doppler imaging was performed with the sample volume at the septal and lateral annulus from the apical 4-chamber view. The frame rate was between 80 and 100 frames per second. All measurements were measured by the same operator and the measurements were averaged over three cardiac cycles and adjusted by the RR interval.

### Statistical analysis

Clinical characteristics are presented as mean and standard deviation (SD) for continuous data and frequency and percentage for categorical data.

Bivariable associations between clinical characteristics, physical activity, self-motivation and self-empowerment with high VO_2_ versus low VO_2_ was first examined by t test or chi squared tests. Variables that were non-normal such as age, blood pressure and body mass index were examined by Mann-Whitney test. High VO_2_ was defined as VO_2_ > 35 (ml/kg/min) for men or VO_2_ > 27 (ml/kg/min) for women as mean VO_2_ was 35 (ml/kg/min) for men and 27 (ml/kg/min) for women in our cohort [22]. Low VO_2_ was defined as VO_2_ ≤ 35 (ml/kg/min) for men or VO_2_ ≤ 27 (ml/kg/min) for women.

Univariate logistic regression was then used to assess the association of self-motivation and self-empowerment with VO_2_. Subsequently, multivariate regression was conducted to determine the role of self-motivation and self-empowerment factors that show an association of *p* < 0.05 with high VO_2_ in univariate analysis adjusting for significant clinical characteristics (age, body mass index, education level). Waist circumference was not used in multivariate adjustment as it was used to estimate VO_2_.

We then examined the relationship between gender and psychosocial determinants of self-motivation and self-empowerment using a chi-squared test. Binary logistic regression was performed to assess the association between gender and self-motivation and self-empowerment factors that had *p* < 0.05 in the chi-squared test adjusting for education level. Low educational level was defined as absence of formal education.

All statistical analysis was conducted using SPSS. For all analyses, a two-tailed *p* value of < 0.05 was considered significant.

## Results

The baseline clinical and demographic characteristics of the study population are presented in Table 1. In total, 180 participants were studied, of which 79 (43.9%) had high VO_2_ and 101 (56.1%) had low VO_2_. Cardiovascular risk factors of hypertension (61.1%), dyslipidemia (58.3%) and diabetes mellitus (22.2%) were found to be prevalent in the study population.

**Table 1:** Comparison of baseline clinical characteristics between low VO_2_ and high VO_2_ groups

	VO_2_ low (*n* = 101)	VO_2_ high (*n* = 79)	Total (*n *= 180)	*p* value
Age (year)	78 (74–80)	73 (69–78)	77 (71–80)	**< 0.0001**
Female	45 (44.6%)	33 (41.8%)	78 (43.3%)	0.763
Weight (kg)	61.5 (55.3–68.7)	60 (50.0–64)	60.4 (54.35–67.6)	0.014
Height (cm)	160 (153-165.2)	159.5 (154–166)	160 (153.5–166)	0.71
Body mass index (kg/m^2^)	24.2 (22.6–26.5)	22.8 (21.0-24.5)	23.7 (21.9–25.6)	**0.0003**
Waist circumference (cm)	90 (86–87)	86 (78–89)	88 (83–93)	< 0.0001
Systolic blood pressure (mmHg)	144 (129–160)	146 (130–156)	145.5 (129-156.5)	0.67
Diastolic blood pressure (mmHg)	76 (69–87)	76 (68–83)	76 (68–85)	0.73
Hypertension	66 (65.3%)	44 (55.7%)	110 (61.1%)	0.219
Dyslipidemia	63 (62.4%)	42 (53.2%)	105 (58.3%)	0.227
Diabetes Mellitus	25 (24.8%)	15 (19%)	40 (22.2%)	0.373
Ever smoker	17 (16.8%)	14 (17.7%)	31 (17.2%)	0.99
Ever drinker	11 (10.9%)	6 (7.59%)	17 (9.4%)	0.331
**Physical Activity: Frequency**				
a. Inactive	27 (26.7%)	2 (2.5%)	29 (16.1%)	
b. Once a week	1 (1%)	7 (8.9%)	8 (4.44%)	
c. 2 to 3 times a week	13 (12.9%)	13 (16.5%)	26 (14.4%)	
d. Almost everyday	60 (59.4%)	57 (72.2%)	117 (65%)	
**Physical Activity: Intensity**				
a. Take it easy	97 (96%)	29 (36.7%)	126 (70%)	
b. Heavy breath and sweat	4 (4%)	48 (60.8%)	52 (28.9%)	
c. Near exhaustion	0 (0%)	2 (2.5%)	2 (1.11%)	
**Physical Activity: Duration**				
a. < 15 min	38 (37.6%)	7 (8.9%)	45 (25%)	
b. 16 to < 30 min	22 (21.8%)	12 (15.2%)	34 (18.9%)	
c. 30 to 60 min	29 (28.7%)	23 (29.1%)	52 (28.9%)	
d. > 1 hour	12 (11.9%)	37 (46.8%)	49 (27.2%)	

Compared to participants in the high VO_2_ subgroup, those with low VO_2_ were found to be older (73(69–78) vs 78(74–80), *p* < 0.0001), had a greater weight (60 (50–64) vs 61.5 (55.3–68.7), *p* = 0.014) and higher BMI (22.8 (21-24.5) vs 24.2 (22.6–26.5), *p* = 0.0003). Those with low VO_2_ were also more likely to have lower levels of education (*p* = 0.049).

Participants with low VO_2_ were less likely than those with high VO_2_ to feel satisfied after exercise (70.3% vs 86.1%, OR 0.38, 95%CI 0.15–0.96, adjusted *p* = 0.041). Across both groups, the top four barriers to regular exercise were lack of time (27.8%), tiredness (26.7%), not being able to afford exercise (12.8%) and pain while exercising (12.2%). The top four barriers to regular exercise were then collapsed and termed “Major Barriers” and were found to be significantly associated with VO_2_ before adjustment (*p* *=* 0.006). After adjustment for age, BMI and education level, participants with high VO_2_ were more likely to express major barriers to exercise (88% vs 72%, *p* = 0.017) (Table 2).

**Table 2:** Comparison of self-motivation and self-empowerment responses between low VO_2_ and high VO_2_ groups

	VO_2_ low (*n* = 101)	VO_2_ high (*n* = 79)	Total (*n* = 180)	*p* value	Adjusted *p* value^a^
**Self-Motivation Questionnaire**					
** I enjoy exercise**				0.081	
a. Not true at all	8 (7.9%)	4 (5.1%)	12 (6.67%)		
b. Moderately true	46 (45.5%)	25 (31.6%)	71 (39.4%)		
c. Very true	47 (46.5%)	50 (63.3%)	97 (53.9%)		
** I exercise because**:				0.314	
a. Improve physical appearance	2 (2%)	0 (0%)	2 (1.11%)		
b. Lose weight	9 (8.9%)	3 (3.8%)	12 (6.67%)		
c. Continue enjoyed activities	28 (27.7%)	24 (30.4%)	52 (28.9%)		
d. Maintain independence	62 (61.4%)	52 (65.8%)	114 (63.3%)		
** I have no time to exercise**				0.305	
a. Not true at all	83 (82.2%)	71 (89.9%)	154 (85.6%)		
b. Moderately true	13 (12.9%)	7 (8.9%)	20 (11.1%)		
c. Very true	5 (5%)	1 (1.3%)	6 (3.33%)		
** I feel exercising will impact chronic diseases**				0.483	
a. Not true at all	12 (11.9%)	6 (7.6%)	18 (10%)		
b. Moderately true	24 (23.8%)	16 (20.3%)	40 (22.2%)		
c. Very true	65 (64.4%)	57 (72.2%)	122 (67.8%)		
** I feel good after exercise**				0.026	**0.041**
a. Not true at all	4 (4%)	1 (1.3%)	5 (2.78%)		
b. Moderately true	26 (25.7%)	10 (12.7%)	36 (20%)		
c. Very true	71 (70.3%)	68 (86.1%)	139 (77.2%)		
** I am confident to participate in regular exercise at least 3 times a week for 20 mins each**				0.175	
a. Not confident	29 (28.7%)	14 (17.7%)	43 (23.9%)		
b. Moderately confident	31 (30.7%)	24 (30.4%)	55 (30.6%)		
c. Very confident	41 (40.6%)	41 (51.9%)	82 (45.6%)		
** My barriers to regular exercise**				0.280	
a. Do not enjoy	9 (8.9%)	1 (1.3%)	10 (5.56%)		
b. Bored with program	7 (6.9%)	3 (3.8%)	10 (5.56%)		
c. Pain when exercising	12 (11.9%)	10 (12.7%)	22 (12.2%)		
d. Tired	23 (22.8%)	25 (31.6%)	48 (26.7%)		
e. Stressed	1 (1%)	2 (2.5%)	3 (1.67%)		
f. No time	26 (25.7%)	24 (30.4%)	50 (27.8%)		
g. Too old	6 (5.9%)	1 (1.3%)	7 (3.89%)		
h. Do not need to	3 (3%)	1 (1.3%)	4 (2.22%)		
i. Do not know how to	2 (2%)	1 (1.3%)	3 (1.67%)		
j. Cannot afford to	12 (11.9%)	11 (13.9%)	23 (12.8%)		
k. Major barriers^b^	73 (72.3%)	70 (88.6%)	143 (79.4%)	0.006	**0.017**
** Factors that motivate me to exercise**				0.527	
a. Family or friends exercise together	17 (16.8%)	7 (8.9%)	24 (13.3%)		
b. Family or friends comment on appearance	3 (3%)	4 (5.1%)	7 (3.89%)		
c. Family or friends help with chores/work	1 (1%)	1 (1.3%)	2 (1.11%)		
d. Instructor to guide exercise	11 (10.9%)	7 (8.9%)	18 (10%)		
e. Able to control type and difficulty of exercise	69 (68.3%)	60 (75.9%)	129 (71.7%)		
**Self-Empowerment Questionnaire**					
** Level of education**					
a. No formal education	18 (17.8%)	6 (7.6%)	24 (13.3%)	0.049	
b. Primary education	34 (33.7%)	22 (27.8%)	56 (31.1%)		
c. Secondary education	49 (48.5%)	51 (64.6%)	100 (55.6%)		
** Smartphone user**	78 (77.2%)	71 (89.9%)	149 (82.8%)	0.029	0.128
** Used smartphone apps to manage chronic diseases**	31 (30.7%)	32 (40.5%)	63 (35%)	0.171	
** Willing to use smartphone apps to manage chronic diseases**				0.013	0.931
a. Yes	52 (51.5%)	45 (57%)	97 (53.9%)		
b. Not sure	3 (3%)	10 (12.7%)	13 (7.22%)		
c. No	46 (45.5%)	24 (30.4%)	70 (38.9%)		
** Factors that encourage or discourage use of smartphone apps to manage health**				0.474	
a. Do not intend to use smartphone apps	19 (18.8%)	8 (10.1%)	27 (15%)		
b. App guides exercise routines	19 (18.8%)	19 (24.1%)	38 (21.1%)		
c. Ability to share feedback with healthcare professionals through app	10 (9.9%)	11 (13.9%)	21 (11.7%)		
d. Reveal of personal information on app	28 (27.7%)	23 (29.1%)	51 (28.3%)		
e. Financial incentives	25 (24.8%)	18 (22.8%)	43 (23.9%)		

^a^Adjusted for age, body mass index, and educational level

^b^Major barriers include having no time, tiredness, pain, and inability to afford exercise

Although a large proportion of both groups used smartphones, those with low VO_2_ were less likely to be smartphone users (77.2% vs 89.9%, *p* = 0.029), although this may have been influenced by baseline educational levels (adjusted *p* = 0.128). Major factors that motivated the use of smartphone applications to manage individual health were financial incentives (23.9%) and guidance on exercise routines (21.1%) while the reveal of personal information was a major deterrent (28.3%).

**Fig. 1:**
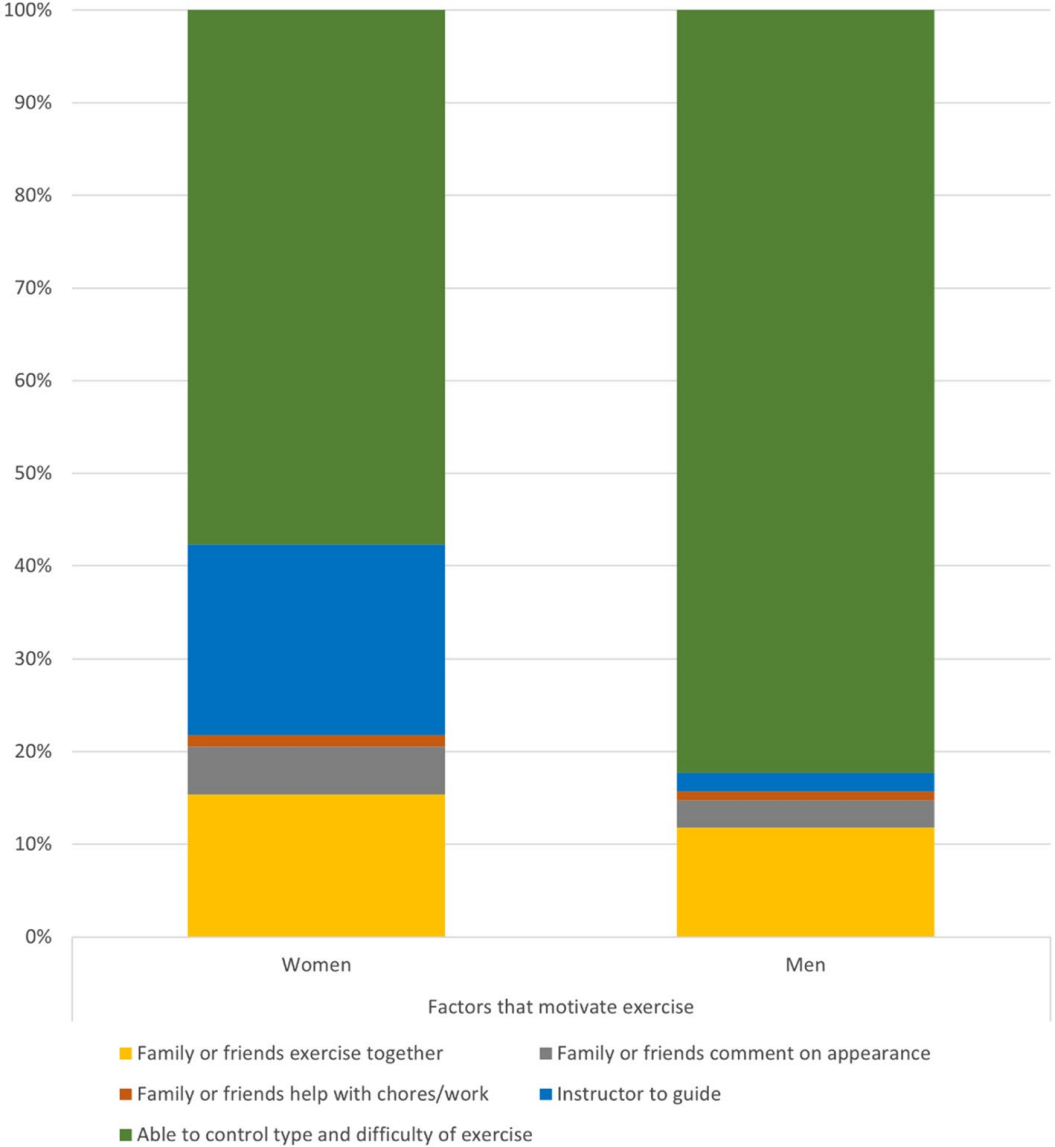
Differences in self-motivation factors between the women and men: Women were more likely than men to be motivated to exercise if they had an instructor to guide them (blue cells: 20.5% vs 1.96%, adjusted *p* = 0.027), whereas men were more likely to participate if they were able to control the type and difficulty of the exercise (green cells: 82.4% vs 57.7%, adjusted *p* = 0.001).

In this study population, there were 78 female participants and 102 male participants. More women had no formal education compared to men (20.5% vs 7.84%, *p* = 0.016). (Table 3). In terms of self-motivation, notable differences in factors that encourage participation in exercise were found between the two genders. Adjusting for education level, a greater proportion of women were more likely to engage in exercise if they had an instructor to guide them (20.5% vs 1.96%, *p* = 0.027) whereas men were more likely to participate if they were able to control the type and difficulty of the exercise (82.4% vs 57.7%, *p* = 0.001) (Fig. 1).

**Table 3:** Gender-based Differences in self-motivation and self-empowerment responses

	Female (*n* = 78)	Male (*n* = 102)	Total (*n* = 180)	*p* value	Adjusted *p* value^a^
**Self-Motivation**					
** I enjoy exercise**				0.135	
a. Not true at all	8 (10.3%)	4 (3.92%)	12 (6.67%)		
b. Moderately true	33 (42.3%)	38 (37.3%)	71 (39.4%)		
c. Very true	37 (47.4%)	60 (58.8%)	97 (53.9%)		
** I exercise because**:				0.531	
a. Improve physical appearance	1 (1.28%)	1 (0.980%)	2 (1.11%)		
b. Lose weight	7 (8.97%)	5 (4.90%)	12 (6.67%)		
c. Continue enjoyed activities	19 (24.4%)	33 (32.4%)	52 (28.9%)		
d. Maintain independence	51 (65.4%)	63 (61.8%)	114 (63.3%)		
** I have no time to exercise**				0.732	
a. Not true at all	66 (84.6%)	88 (86.3%)	154 (85.6%)		
b. Moderately true	10 (12.8%)	10 (9.80%)	20 (11.1%)		
c. Very true	2 (2.56%)	4 (3.92%)	6 (3.33%)		
** I feel exercising will impact chronic diseases**				0.493	
a. Not true at all	10 (12.8%)	8 (7.84%)	18 (10%)		
b. Moderately true	18 (23.1%)	22 (21.6%)	40 (22.2%)		
c. Very true	50 (64.1%)	72 (70.6%)	122 (67.8%)		
** I feel good after exercise**				0.141	
a. Not true at all	4 (5.13%)	1 (0.980%)	5 (2.78%)		
b. Moderately true	18 (23.1%)	18 (17.6%)	36 (20%)		
c. Very true	56 (71.8%)	83 (81.4%)	139 (77.2%)		
** I am confident to participate in regular exercise at least 3 times a week for 20 mins each**				0.886	
a. Not confident	20 (25.6%)	23 (22.5%)	43 23.9%)		
b. Moderately confident	23 (29.5%)	32 (31.4%)	55 (30.6%)		
c. Very confident	35 (44.9%)	47 (46.1%)	82 (45.6%)		
** My barriers to regular exercise**				0.177	
a. Do not enjoy	6 (7.69%)	4 (3.92%)	10 (5.56%)		
b. Bored with program	2 (2.56%)	8 (7.84%)	10 (5.56%)		
c. Pain when exercising	13 (16.7%)	9 (8.82%)	22 (12.2%)	0.111	
d. Tired	18 (23.1%)	30 (29.4%)	48 (26.7%)	0.341	
e. Stressed	2 (2.56%)	1 (0.980%)	3 (1.67%)		
f. No time	25 (32.1%)	25 (24.5%)	50 (27.8%)	0.263	
g. Too old	1 (1.28%)	6 (5.88%)	7 (3.89%)		
h. Do not need to	1 (1.28%)	3 (2.94%)	4 (2.22%)		
i. Do not know how to	2 (2.56%)	1 (0.980%)	3 (1.67%)		
j. Cannot afford to	8 (10.3%)	15 (14.7%)	23 (12.8%)	0.376	
k. Major barriers^b^	64 (82.1%)	79 (77.5%)	143 (79.4%)	0.713	
**Factors that motivate me to exercise**					
a. Family or friends exercise together	12 (15.4%)	12 (11.8%)	24 (13.3%)	0.479	
b. Family or friends comment on appearance	4 (5.13%)	3 (2.94%)	7 (3.89%)	0.452	
c. Family or friends help with chores/work	1 (1.28%)	1 (0.980%)	2 (1.11%)	0.848	
d. Instructor to guide exercise	16 (20.5%)	2 (1.96%)	18 (10%)	< 0.0001	**0.027**
e. Able to control type and difficulty of exercise	45 (57.7%)	84 (82.4%)	129 (71.7%)	< 0.001	**0.001**
**Self-Empowerment**					
** Level of education**				0.002	
a. No formal education	16 (20.5%)	8 (7.84%)	24 (13.3%)		
b. Primary education	30 (38.5%)	26 (25.5%)	56 (31.1%)		
c. Secondary education	32 (41.0%)	68 (66.7%)	100 (55.6%)		
** Smartphone user**	58 (74.4%)	91 (89.2%)	149 (82.8%)	0.01	**0.039**
** Used smartphone apps to manage chronic diseases**	25 (32.1%)	38 (37.3%)	63 (35%)	0.468	
** Willing to use smartphone apps to manage chronic diseases**				0.164	
a. Yes	40 (51.3%)	57 (55.9%)	97 (53.9%)		
b. Not sure	3 (3.85%)	10 (9.80%)	13 (7.22%)		
c. No	35 (44.9%)	35 (34.2%)	70 (38.9%)		
** Factors that encourage or discourage use of smartphone apps to manage health**				0.002	
a. Do not intend to use smartphone apps	19 (24.4%)	8 (7.84%)	27 (15%)	0.003	**0.01**
b. App guides exercise routines	8 (10.3%)	30 (29.4%)	38 (21.1%)	0.002	**0.001**
c. Ability to share feedback with healthcare professionals through app	8 (10.3%)	13 (12.7%)	21 (11.7%)	0.647	
d. Reveal of personal information on app	21 (26.9%)	30 (29.4%)	51 (28.3%)	0.741	
e. Financial incentives	22 (28.2%)	21 (20.6%)	43 (23.9%)	0.290	

^a^Adjusted for age, body mass index, and educational level

^b^Major barriers include having no time, tiredness, pain, and inability to afford exercise

In terms of barriers to regular exercise participation, both men and women reported similar barriers. Women were more likely to report a lack of time (32.1% vs 24.5%, *p* = 0.263) and pain when exercising (16.7% vs 8.82%, *p* = 0.111) whereas men were more likely to report tiredness (29.4% vs 23.1%, *p* = 0.341) and inability to afford exercise (14.7% vs 10.3%, *p* = 0.376) **(**Table 3). Nevertheless, both groups reported similar barriers, of which the top four were lack of time, tiredness, inability to afford exercise and pain when exercising.

**Fig. 2:**
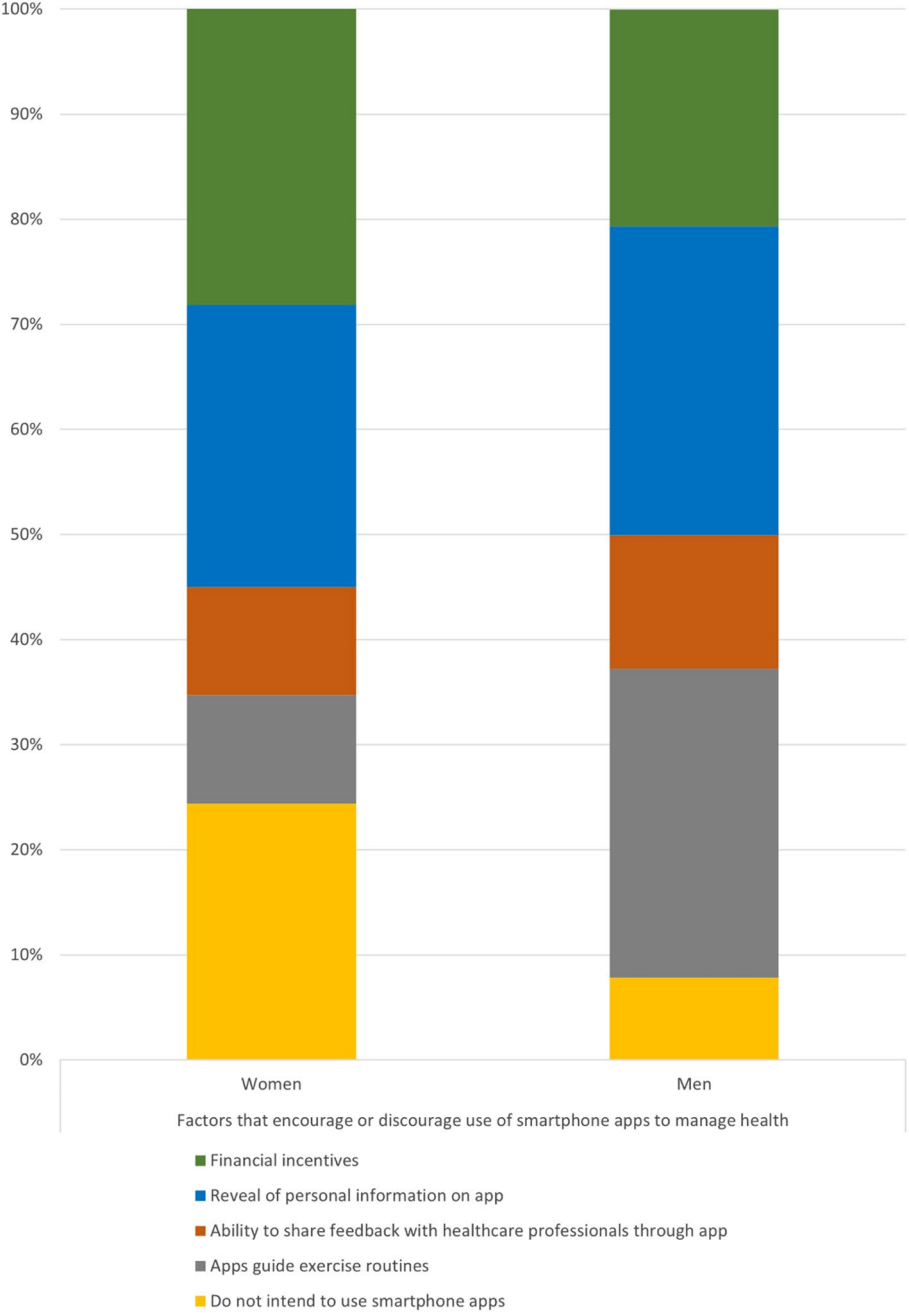
Gender-based differences in factors affecting the use of smartphone applications for management of health conditions: A larger proportion of women did not intend to use smartphone applications to manage health (yellow cells: 24.4% vs 7.84%, adjusted *p* *=* 0.01). Men were more likely to use applications if it could also guide them on exercise routines (grey cells: 29.4% vs 10.3%, adjusted *p* *=* 0.001).

Despite adjusting for differences in education, levels of smartphone use remained lower among women compared to men (74.4% vs 89.2%, adjusted for education *p* = 0.039). However, there were distinct differences in smartphone preferences between the genders. Despite adjustments for education level, we observed significant differences in factors that encouraged or discouraged the use of smartphone applications to manage health between the genders (Fig. 2). The largest differences between men and women were observed in two factors: unwillingness to use smartphone applications and application-guided exercise routines. Women were more unwilling to utilize smartphone applications (24.4% vs 7.84%, OR 3.3, 95%CI 1.34–8.19, adjusted *p* = 0.01) whereas men were more encouraged to use smartphone applications to manage health if the applications could guide them on exercise routines (29.4% vs 10.3%, adjusted *p* = 0.001).

## Discussion

Among a community-based sample of older adults, factors associated with high aerobic capacity were higher educational status and a sense of feeling good after exercise. Across levels of aerobic capacity, both groups expressed major barriers to exercise, which included pain after exercise, lack of energy to exercise, financial affordability for exercise, and lack of time.

Our observations regarding factors that promote regular exercise, such as post-exercise sense of feeling good, concur with pre-existing studies that have also identified intrinsic motivators such as satisfaction for maintenance of long-term exercise [26, 27]. While this is a subjective measurement, the optimal changes in aerobic capacity associated with feeling good after exercise may reflect actual beneficial changes in the cardiovascular system associated with long-term engagement in exercise [28]. Although the association between education level and aerobic capacity have been previously reported [3], the underlying mechanism may not be due to poor health literacy alone. In our sample, over 80% of those with low aerobic capacity acknowledged the beneficial effects of exercise on chronic health conditions. This may suggest a high level of awareness of exercise and its health benefits among the sampled population. Instead, other factors related to education level such as socio-economic status may reduce time available for leisure-time physical activity[18, 29]. This reflects a lack of means rather than lack of knowledge as a factor associated with lower aerobic capacity. This implies that future implementation strategies should address a lack of means to exercise, rather than just raising awareness.

In terms of barriers to regular exercise, participants in the high VO_2_ group expressed more barriers to regular exercise. This could reflect inherently higher levels of exercise participation among those in the high VO_2_ group, resulting in greater response rates to these questions. Other unidentified barriers not obtained through our current questionnaire may explain the poor uptake of regular exercise among those with low VO_2_ [30].

Both genders expressed similar barriers to exercise. In aggregate, barriers to exercise such as pain after exercise, lack of energy to exercise, financial affordability for exercise, and lack of time, accounted for over three-quarters of these barriers among both genders. Most of these barriers were also identified in previous studies [31, 32]. From a preventive health standpoint, solutions that target these major barriers may improve exercise participation in the community.

For example, our study identified key gender differences in self-motivation and self-empowerment factors related to exercise. Men in our study were more motivated to exercise if they could control the type and difficulty of exercise they engaged in, suggesting that men desired greater independence in terms of exercise. This could be due to men possessing more intrinsic motivators such as being competitive [33], thus challenging themselves to engage in exercise to progressively improve in terms of strength and mastery. On the other hand, women in our study were more likely to exercise if they had an instructor to guide them. This could suggest that women preferred more structured and guided forms of exercise, which concurs with a prior study that also found that women were more likely to participate in organized exercise programs than men [34]. Given that instructor-led programs are also more likely to be conducted in groups, our results also lend support to previous studies which suggest that women experience greater satisfaction from exercising in groups due to the support from fellow individuals [35]. Tailoring exercises to meet these gender-specific expectations such as group-based exercises for women or greater autonomy given to men to dictate their own exercise, may possibly enhance exercise satisfaction and hence increase intrinsic motivation to participate in leisure-time physical activity.

As a contemporary current study, the data on smartphone usage and its associations with aerobic capacity among older adults is likely insightful in line with increasing digitalization of healthcare [36]. Our data confirm high rates of smartphone usage in our community, although these observations regarding smartphone use may be an accompanying social factor rather than a causal factor, one that is closely related to underlying educational levels among those with high aerobic capacity and among men. In certain societies, cultural biases may exist where women take on domestic roles [37] more often than men, lowering their overall need for a smartphone.

Interestingly, only a quarter of the cohort felt that financial factors such as cash vouchers or discounts would incentivize their use of smartphone health applications. This was observed to be similar between high versus low aerobic capacity groups and additionally between women and men. Our observations are in line with a recently published trial by Yamashita et al [38] who studied whether financial incentives alone versus financial incentives plus social network incentives could lead to changes in physical activity. The authors found that financial incentives alone did not significantly improve physical activity within a three-month period, among a small sample of 39 older women aged 65 years and above. Concurring with their findings, our current data that involves a larger sample of older adults consisting of both genders, suggests a limited role for the use of financial incentives in exercise programs, in relation to digital health applications.

On the other hand, targeted measures tailored to gender-based preferences may be a better strategy for smartphone-based exercise applications. For men, inclusion of instructions to guide exercise in smartphone applications is a possible strategy to promote exercise among men, who may be driven by greater desire for self-efficacy [39]. Conversely, a large portion of women in our study did not intend to utilize smartphone applications. This may once again reflect pre-existing cultural beliefs regarding the role of women in a family.

We acknowledge limitations in the present study. Firstly, we had used a validated calculator of peak oxygen uptake to estimate aerobic capacity among these community adults. Aerobic capacity in these older participants may be influenced by underlying age-related deteriorations in cardiovascular health, such that comparisons in psychosocial factors based on aerobic capacity may inadequately reflect psychosocial factors that determine participation in exercise. However, because high VO_2_ in this cohort was previously validated in association with better cardiovascular function [22], current comparisons based on this definition of VO_2_ will likely encapsulate inherent biological differences between the groups. This allows for future strategies that target differences in psychosocial factors gleaned out between these two groups to be more clinically impactful. Secondly, a more granular measure of physical activity encompassing a range of activities may capture motivations between types of physical activity otherwise not identified by our questionnaires. Future studies could address this limitation by more systematic measurement of specific activities and the level at which participants are engaged. Thirdly, our observations regarding smartphone applications were contextualized among older adults and may not be applicable to younger adults, for secondary prevention of diseases or among other ethnicities. However, in the field of cardiovascular disease management where increases in smartphone application use have been associated with improvements in risk factor management (such as weight and body mass index), our observations provide important insights into how future studies may enhance application user engagement [40]. Our study did not involve an evaluation of cognitive function or pre-existing history of mental illnesses. As participants had to sign informed consent prior to participating in the study, it is unlikely that mental incapacity is a concern in this study. However underlying conditions such as undiagnosed depression or subtle cognitive impairments may still exist and influence their psychosocial responses [18]. The use of concurrent medications may influence physical activity practices or dietary habits but were not included in the present analyses. Finally, the sample size was not prespecified to detect gender differences in smartphone usage and psychosocial responses, hence a larger sample may unveil more differences. This analysis consisted of recruited participants up till 2020 due to the coronavirus pandemic that had interrupted further recruitment.

Despite these limitations, our study highlighted key psychosocial factors that may influence the practice of exercise among community older adults. Future studies targeting these factors may impact cardiovascular health strategies for older adults.

## Conclusion

Among community older adults, factors associated with high aerobic capacity were determinants of self-motivation such as a sense of feeling good after exercise. Across levels of aerobic capacity, both groups expressed major barriers to exercise, which included pain after exercise, lack of time to exercise, financial affordability for exercise, and lack of time. Older adults with high aerobic capacity were more likely to be smartphone users and expressed willingness to use smartphone applications to manage chronic disease. Women preferred fitness instruction but not via smartphone apps, while men preferred guided exercise routines via smartphone applications. Focusing on psychosocial factors and gender-based patterns in exercise may improve exercise participation in older adults.

## Data Availability

The datasets generated and/or analysed during the current study are not publicly available due to institutional restrictions but are available from the corresponding author on reasonable request.
